# Special architecture and anti-wear strategies for giant panda tooth enamel: Based on wear simulation findings

**DOI:** 10.3389/fvets.2022.985733

**Published:** 2022-09-14

**Authors:** Yuanheng Wu, Jinxing Liu, Yongqiang Yang, Shaotong Tu, Zichen Liu, Yingyun Wang, Chen Peng, Gang Liu, Yipeng Jin

**Affiliations:** ^1^Department of Clinical Veterinary Medicine, College of Veterinary Medicine, China Agricultural University, Beijing, China; ^2^Tsinghua Laboratory of Brain and Intelligence, Nonhuman Primate Research Center Tsingua University, Beijing, China; ^3^Department of Preventive Veterinary Medicine, College of Veterinary Medicine, China Agricultural University, Beijing, China

**Keywords:** giant pandas, conservation animal, tooth enamel, microcratch, wear resistance

## Abstract

Giant pandas are the flagship species in world conservation. Due to bamboo being the primary food source for giant pandas, dental wear is common owing to the extreme toughness of the bamboo fiber. Even though research on tooth enamel wear in humans and domestic animals is well-established, research on tooth enamel wear in giant pandas is scarce. The purpose of this study is to evaluate tooth enamel wear resistance in giant pandas to provide a basis for a better understanding of their evolutionary process. From microscopic and macroscopic perspectives, the abrasion resistance of dental enamel in giant pandas is compared with that of herbivorous cattle and carnivorous dogs in this study. This involves the use of micro-scratch and frictional wear tests. The results show that the boundary between the enamel prism and the enamel prism stroma is well-defined in panda and canine teeth, while bovine tooth enamel appears denser. Under constant load, the tribological properties of giant panda enamel are similar to those of canines and significantly different from those of bovines. Test results show that the depth of micro scratches in giant panda and canine enamel was greater than in cattle, with greater elastic recovery occurring in dogs. Scratch morphology indicates that the enamel substantive damage critical value is greater in pandas than in both dogs and cattle. The analysis suggests that giant panda enamel consists of a neatly arranged special structure that may disperse extrusion stress and absorb impact energy through a series of inelastic deformation mechanisms to cope with the wear caused by eating bamboo. In this study, the excellent wear resistance of giant panda's tooth enamel is verified by wear tests. A possible theoretical explanation of how the special structure of giant panda tooth enamel may improve its wear resistance is provided. This provides a direction for subsequent theoretical and experimental studies on giant panda tooth enamel and its biomaterials.

## Introduction

Mammal tooth morphology is complex. In the evolutionary process, the animal's tooth structure has generally become consistent with the animal's feeding habits and behaviors (1). Therefore, studying teeth is essential to obtaining clues about an animal's dietary, behavioral, and evolutionary history. Carnivores tend to have sharper and more penetrating teeth, which are used as hunting weapons for piercing and tearing the prey. On the other hand, herbivores have harder, more friction-resistant teeth that allow them to grind tough fruits and seeds.

The giant panda is a distinct species in mammalian evolutionary history. Unlike other species in the Carnivora Ursidae family, giant pandas gradually evolved into a predominately exclusively bamboo-eating species during the Pleistocene (2, 3). Through stable isotope analysis, scientists have determined that the modern giant panda has obvious nutritional differentiation from both carnivores and herbivores, indicating a unique nutritional and ecological niche (4). However, the giant panda's oral and digestive systems still retain several carnivore characteristics (5, 6). This contradiction between physiological structure and feeding habits has made the research on giant pandas' feeding behaviors a hot topic.

Like other carnivores, giant pandas have much shorter and simpler intestines than herbivores and can effectively digest bamboo (7, 8), particularly the cellulose in its cell walls. To obtain enough energy from low-protein, high-fiber, and high-lignin bamboo, giant pandas need to consume large amounts of bamboo every day (9), placing huge pressure on their teeth. Consequently, relevant scientists are increasingly focusing their research on the tooth structure of the giant pandas.

Several studies have demonstrated that to adapt to changing food habits, the giant panda's mouth and teeth macrostructures have undergone a variety of changes to increase bite force and masticatory efficiency (5, 10). However, on a microscopic level, this increased occlusal force also increases enamel wear risk (11). Multiple studies have investigated the microstructure and mechanical behavior of giant panda tooth enamel under different conditions (12). However, there is still a lack of intuitive comparison with other herbivores and carnivores.

As a result, in our study, cattle and dogs were selected as examples of herbivores and carnivores, respectively, to explore the friction and wear of giant panda tooth enamel from different aspects, and the tooth enamel performance of all three species was compared and analyzed. We found the evidence to be consistent with the evolutionary process, from the unique characteristics of giant panda tooth enamel and the importance of this structural adaptive change to enable giant pandas to maintain normal eating, longevity, and, subsequently, species persistence.

## Methods and materials

Tooth samples were collected, including 2-year-old beef cattle mandibular incisors from a slaughtered animal, the right maxillary third premolars from a 4-year-old dog who had died of non-oral diseases, and the right maxillary third premolars from a 4-year-old giant panda who died of natural causes in Qingchuan, Sichuan. All teeth were dentally intact and free of caries and were stored in distilled water at 0–4°C before preparation to avoid any enamel cracking due to tooth dehydration.

### Material and sample preparation

The teeth were placed in a digitally controlled ultrasonic cleaner for 5 min to remove dirt on the surface, followed by alternate washing with absolute ethanol and distilled water before being subjected to sample preparation. The tooth roots were removed with a high-speed drill on a dental workbench, and the remaining crowns were used in the experiment. The crown was cut along the far mesial direction and divided into two parts—the lingual side and the buccal side—while keeping the occlusal surface as complete as possible. The labial surface of cattle teeth, the lingual side surface of the giant panda, and the dog's teeth were divided into several samples containing enamel and dentin, as close to flat as possible, for the next embedding operation.

### Embedding

The friction, wear, and micro-scratch tests require a regular sample shape with a smooth surface. Therefore, it was necessary to embed the cut tooth sample into specific shapes. Gypsum was chosen to make the molds due to its hardness, fast formation, and convenience of drawing. Considering the possible influence of contact between the silicon nitride ball, diamond indenter, and tooth material in the tests, we used the hot-melt resin with a light-cured resin. The high hardness of light-cured resin helps to stabilize samples during the tests. Firstly, gypsum was made into a cube groove mold with a length, width, and height of 1 × 1 × 1 cm. In this experiment, hot melt resin was used to pave the lower half of the plaster mold. After cooling and solidification, the upper half was made with light-curing resin, filled in layers, and the teeth were embedded inside the upper half. After filling each layer with light-curing resin, it was then irradiated with a light-curing instrument. After solidification, it was then filled until flush with the edge of the mold. The enameled tooth surface faced upward and was parallel to the bottom surface.

### Polishing

The embedded samples were removed from the gypsum mold, and a high-speed emery card was used to smoothen and polish the surface of the resin until the tooth surface was exposed. A 3M water scrub paper with particle sizes of P400, P800, P1500, and P2000 was used. In addition, the samples were then polished by hand under water cooling. After primary polishing, the sample surface was then further polished with Mudan^®^ diamond. The particle sizes were W14, W10, W5, W3.5, and W1. Using a variable speed polishing machine with a wool polishing head allowed the samples to be polished to the same extent. Finally, two tooth samples of each animal were obtained.

### Micro-scratch test

An Anton Paar Micro-Scratch Tester 3 (MST3) was used in this experiment. The micro-scratch tester has a variety of functions, including an automatic video microscope, an acoustic emission sensor, and other synchronous zoom panoramic technology functions. The scratch depth measurement and automated imaging were performed using front and rear scanning functions, and the load was applied to an automatic force feedback control device. Different diamond tip types were selected according to the requirements. The pressure head slid at a constant speed under a steady or increasing load on the sample surface. When the MST3 was applied, the pressure reached a certain load, and the samples began to undergo a series of changes. The critical values were accurately detected using tangential force, penetration depth, data recorded by acoustic emission sensors, and observations by built-in optical microscopes. The critical load data was quantitatively analyzed using different sensors (acoustic emission, penetration depth, and friction) and video-microscopic observation. A displacement sensor detected the needle surface profile before, during, and after scratching the sample, allowing the penetration depth of the pressure head to be evaluated both during and after sliding. This enabled a more reliable understanding of the scratch and scratch resistance characteristics. An active feedback system was used to ensure repeated scratch tests and to automatically detect the critical load to optimize the results. At the same time, the accurate instrument displacement positioning system realized accurate positioning during the operation process. A Rockwell I-014 diamond spherical indenter pressure head was used in this experiment with a radius of curvature of 10 μm. At the same time, the variable load range was adjusted according to the scratch surface morphology.

Before starting the experiment, each sample was stored at room temperature for more than 12 h to prevent dehydration. First, the cattle and giant panda tooth samples were subjected to a micro-scratch test under variable loads of 30–100 mN with a scratch length of 200 μm and a speed of 400 μm/min. The scratch depth, critical force value of scratch change local characteristics, and microscopic observation results were recorded. The giant panda and dog tooth samples were subjected to a micro–scratch test under a variable load of 30–500 mN. The scratch length was 200 μm with a speed of 400 μm/min. Finally, the cattle tooth sample was subjected to a micro-scratch test under a variable load of 30–1,000 mN with a scratch length of 200 μm and a speed of 400 μm/min. The scratch depth, critical force value of scratch change local characteristics, and microscopic observation results were recorded. A ConScan surface profiler scanning indenter was used to scan the front, middle, and back of the sample surface, and the 3D surface topography was established *via* a grating scanning confocal white profiler. Three scans were performed on the same surface to accurately record the penetration depth (Pd) and the residual depth (Rd) of the scratch. The first pass is known as the pre-scan and records the sample contour; the second pass is called the loaded scan with increasing load and records the Pd value; the third pass records the depth value Rd after the scratch test and is called the post-scan. The pre- and post-scanning were performed using very low contact force so as not to affect the sample surface status. The Rd was defined as the sample surface plastic deformation. The elastic recovery performance was determined by measuring the difference between Pd and Rd. Tooth enamel is an inelastic material; thus, plastic deformation will occur when the diamond indenter comes in contact with the tooth enamel, and sufficient load is applied. The surface plastic deformation was defined as Rd and called permanent scarring, damage, or residual indentation. The Rd maintained its shape and did not recover to its original status after interacting with the load indenter and the material. As shown in Formula 2–1, elastic recovery (Er) is the difference between the sample deformation under load and the permanent plastic deformation:


(1) $$\begin{array}{r} \text{Er}=\left(\text{Pd }-\text{ Rd}\right)/\text{Pd} \end{array}$$

### Friction-wear test

The Rtec MFT-5000 vertical multi-function friction and wear testing machine was employed in this experiment. This machine can simulate a variety of actual working conditions and perform various tribology and mechanical property tests to ascertain friction, wear, and lubrication characteristics. Test parameters including motion direction, duration, velocity, and acceleration can be controlled. The software can collect, display, analyze, and record test parameters in real time, such as force, displacement, wear depth, temperature, and time.

After being stored at room temperature for 12 h, the surfaces of the tooth samples were cleaned with absolute ethanol, dried, and clamped onto the test bench. Tests were performed according to each specific set of experimental parameters. The abrasive employed was a 10 mm diameter silicon nitride (Si_3_N_4_) ball. Artificial saliva with a pH of 6.8 was selected as the lubricant.

Test parameters are as follows:

Load: 20 N
Abrasive: silicon nitride ball
Abrasive diameter: 10 mm
Lubricant: artificial saliva (pH = 6.8)
Stroke: 0.4 mm
Frequency: 2 Hz
Number of cycles: 500

## Results

### Micro-scratch test

**Figure 1:**
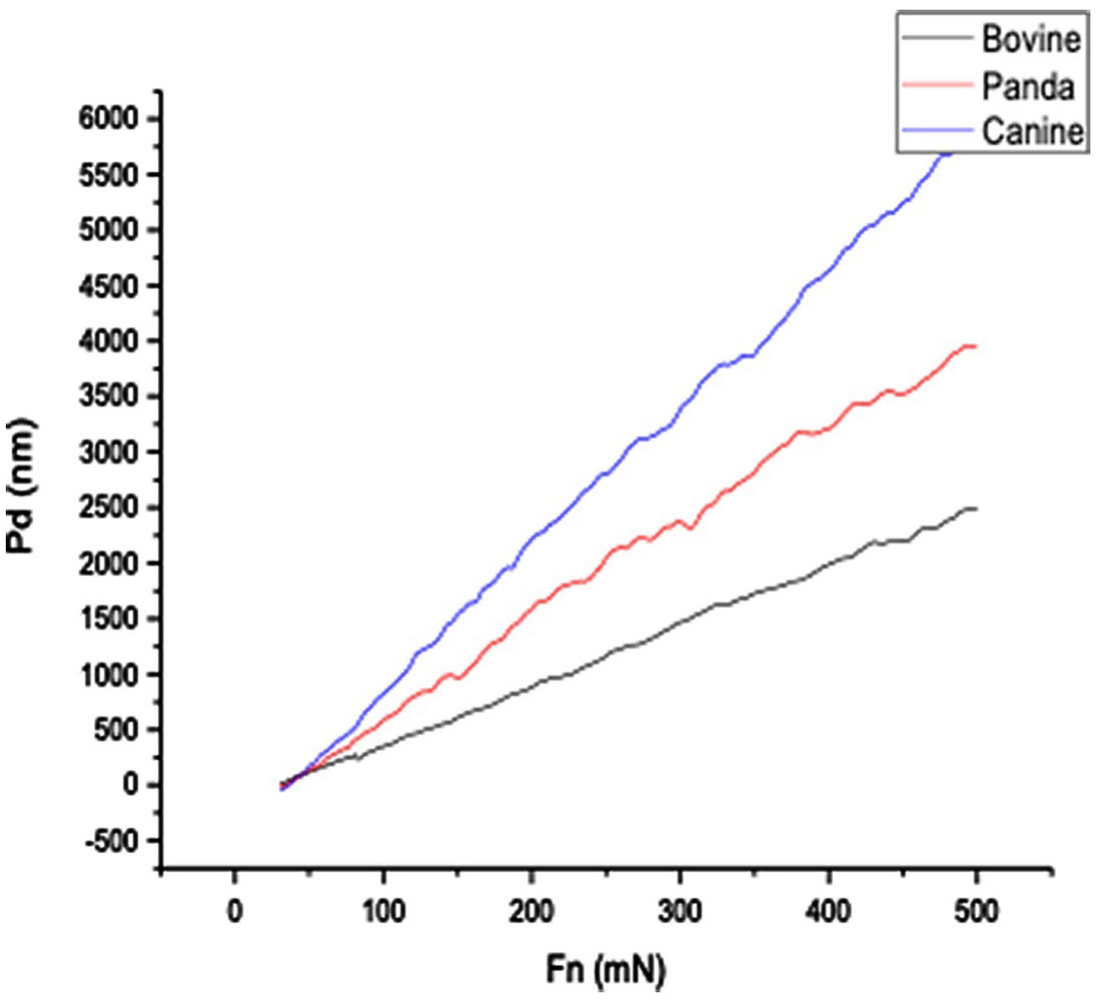
Bovine, giant panda, and canine enamel Pd profile.

**Figure 2:**
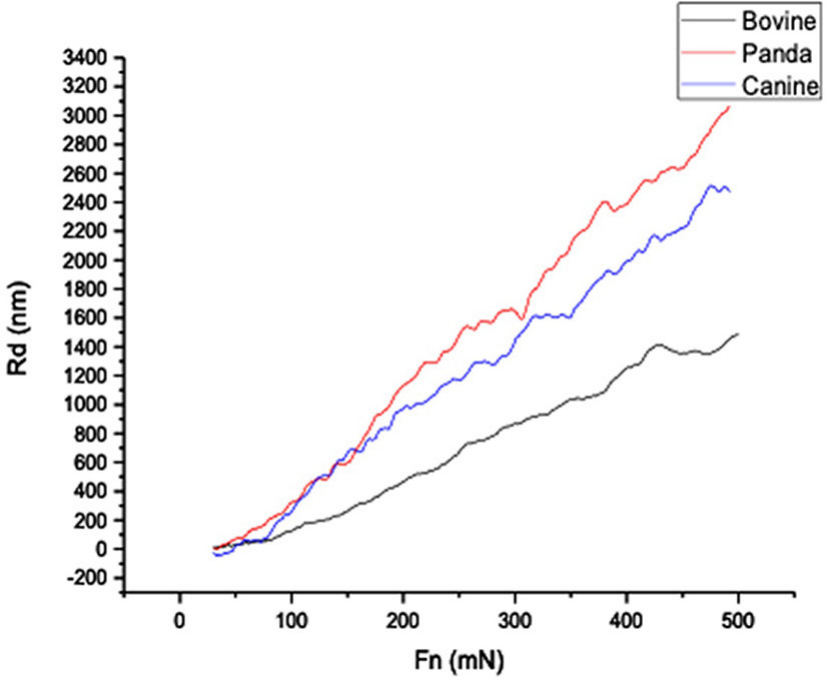
Giant panda and canine enamel Rd profile.

**Figure 3:**
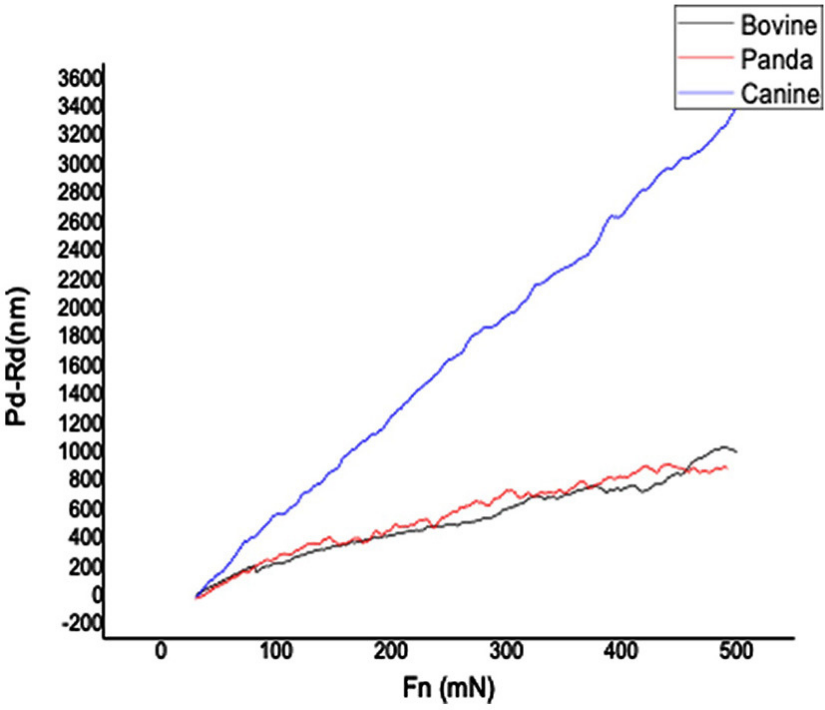
Giant panda and canine enamel Pd-Rd profile.

Figures 1, 2, respectively, show the enamel Pd and Rd curves of the three animals in a loading range of 30–500 mN. It can be seen from Figure 1 that the giant panda's tooth enamel scratch Pd value fell between those of the dog and bovine. The residual depth Rd curve after scratching the surface is shown in Figure 2. The bovine tooth enamel Rd value was the smallest among these animals; however, the Rd value of big panda tooth enamel was greater than that of dogs. Figure 3 shows the difference in curves between Pd and Rd for the three animals. Five hundred twenty-five data sets were calculated and analyzed between 48 and 500 mN using Orign data analysis software. By comparing the PD-RD difference curves, it was found that the difference between the Pd and Rd of giant pandas and cattle was small and far less than that of dogs; that is, the tooth enamel depth change of giant pandas and cattle was similar during and after the micro-scratch test.

**Figure 4:**
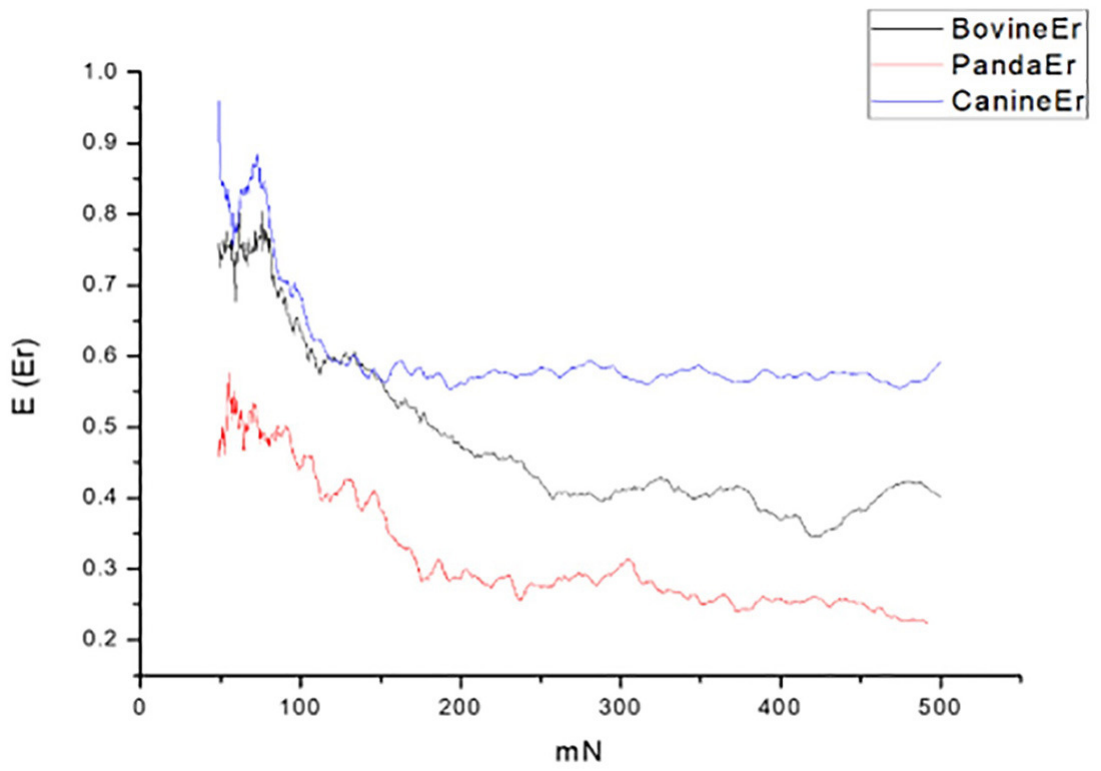
Er curves of cattle, giant panda, and dog enamel.

By comparing the tooth enamel Er value curves of the three animals, it was found that the tooth enamel elastic recovery Er value of giant pandas was always the lowest of the three samples; that is, the giant panda's tooth enamel elastic recovery ability was lower than the other two species, as shown in Figure 4.

### The tooth surface morphology of cattle, giant panda, and dog following a corresponding variable-load scratch test

When scanning the enamel *via* a ConScan surface profiler, the load corresponding to the sudden surface morphology change during the surface scratching is called the critical load (Lc). This can be used to characterize the material's scratch resistance.

**Figure 5:**
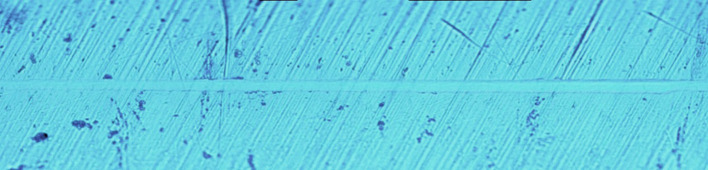
Scratch morphology of bovine enamel under a 30–100 mN load.

**Figure 6:**
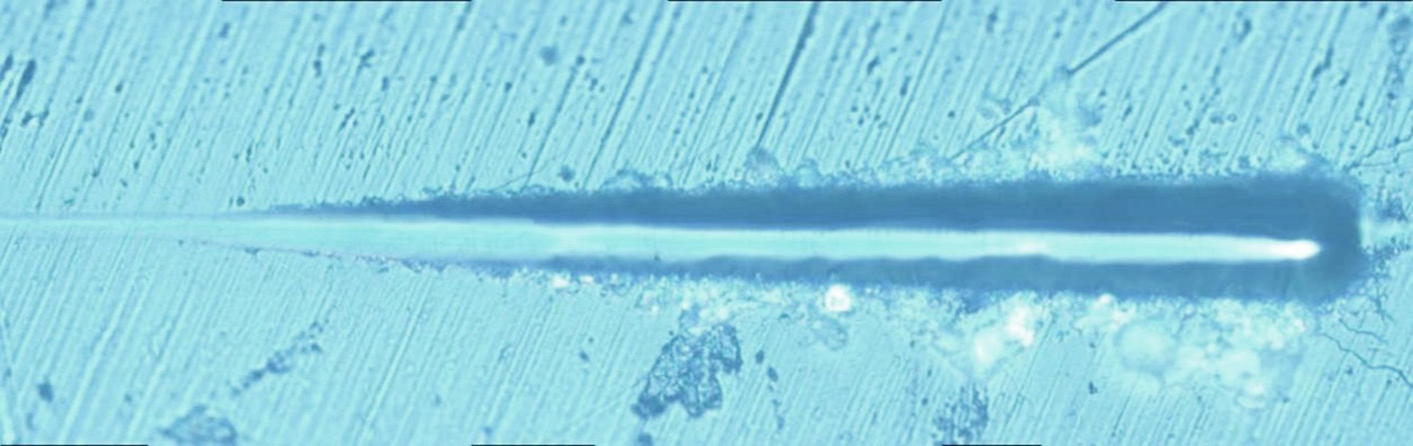
Scratch V of bovine enamel under 80–1,000 mN loading.

**Figure 7:**
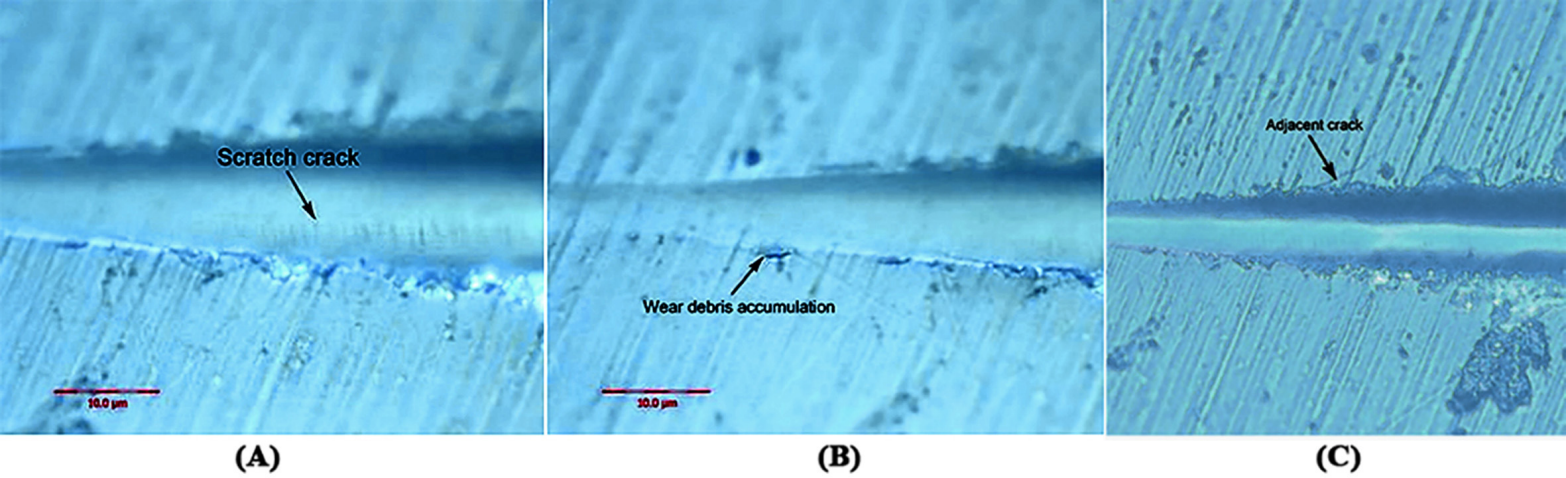
Critical load of bovine tooth enamel morphology change. **(A)** The arrows indicate the scratch crack. **(B)** The arrows indicate wear debris accumulation. **(C)** The arrows indicate adjacent crack.

Figure 5 show that when the load was changed from 30 to 100 mN, the pinpoint caused no obvious scratch cracks or wear debris accumulation on the tooth enamel. Figure 6 shows that obvious wear debris accumulation and scratch cracks occurred in the tooth enamel with a load of 80–1,000 mN. However, there were few adjacent cracks until a larger adjacent crack appeared at the end of the scratch. Figure 7 shows the bovine enamel critical morphology with significant changes. Figure 7B shows that during the scratching of the bovine enamel, the first obvious debris on both sides appeared at 193.78 mN. Figures 7A,B illustrate the onset of scratch cracks at 306.41 mN. Adjacent cracks simultaneously appear on both sides; the number of cracks is small, with the cracks extending linearly.

**Figure 8:**
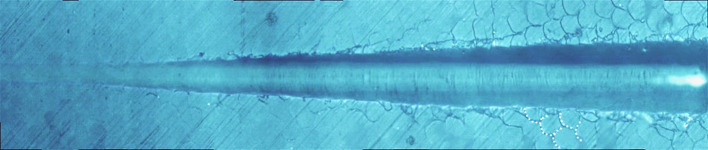
Micro-scratch morphology of giant panda tooth enamel under 30–500 mN varying load. The dotted lines show the shape of the adjacent crack to the enamel column.

**Figure 9:**
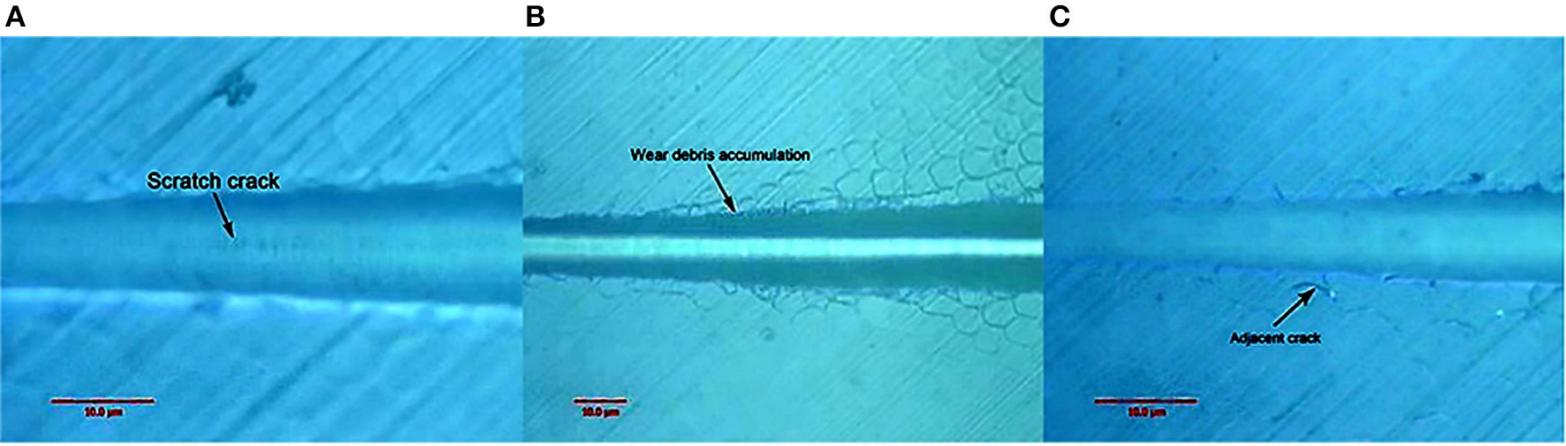
Surface morphology of giant panda tooth enamel under critical load. Arrows in the figure indicate the critical position. **(A)** The arrows indicate the scratch crack. **(B)** The arrows indicate wear debris accumulation. **(C)** The arrows indicate adjacent crack.

Figure 8 shows the giant panda's tooth enamel micron scratch morphology under a variable loading range of 30–500 mN. A small amount of debris accumulated on both sides of the scratches, with dense adjacent cracks consistent with the shape of the enamel column, as shown in Figure 9. This shows the noticeable morphological micro scratches in the tooth enamel. During the giant panda tooth enamel scratching, adjacent cracks first appeared on both sides at the site of 118.85 mN (Figure 9C). The crack shape was generally regular and similar to the shape of the enamel columns. Subsequently, a scratch crack appeared at 271.58 mN (Figure 9A), and, finally, wear debris accumulation appeared at 334.72 mN (Figure 9B).

**Figure 10:**
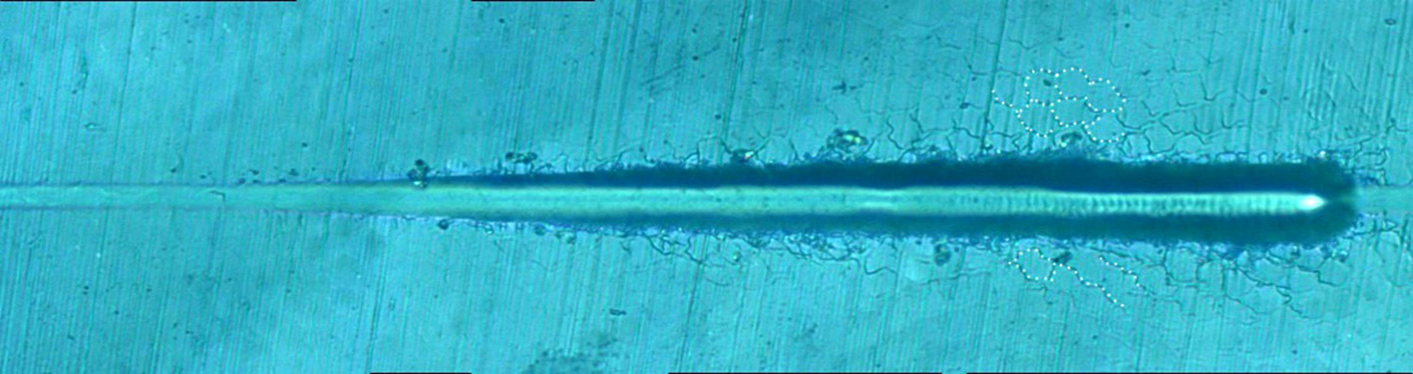
Micro-scratched morphology of canine enamel under a 30–500 mN load. Adjacent crack profiles are consistent with the shape of the enamel column.

**Figure 11:**
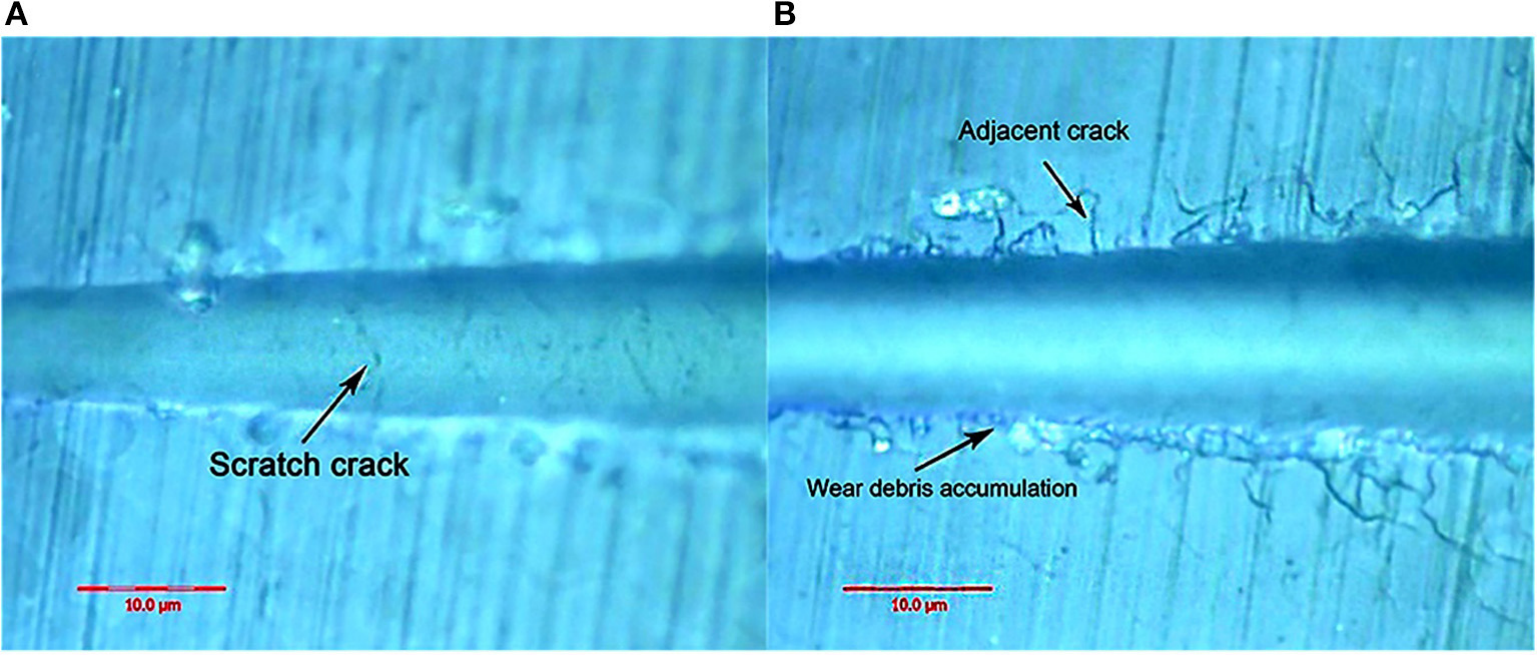
Surface morphology of canine enamel at critical load. **(A)** The arrows indicate the scratch crack. **(B)** The arrows indicate wear debris accumulation and adjacent crack, respectively.

Figure 10 shows the canine enamel micron scratch morphology in a variable load range of 30–500 mN. There was significant debris accumulation on both sides of the scratches, with the connecting cracks being a regular shape, which is consistent with the enamel column. Figure 11 shows the canine enamel micron scratch morphology with significant changes. The first scratch crack appeared at 181.81 mN (Figure 11A), followed almost simultaneously by adjacent cracks and the appearance of debris accumulation at 222.47 mN (Figure 11B).

From the first contact between the pressure head and the enamel surface to the maximum load, the enamel surface was the first to undergo elastic deformation as the scratch load increased, followed by plastic flow with zero wear. Due to the pressure head extrusion, the enamel surface appeared on both sides of the uplift, forming grooves, but there was no substantive damage. When the load exceeded the enamel's elastic deformation limit, plastic deformation occurred, with the surface exhibiting plastic flow traces. As the load continued to increase, the brittle enamel surface fractured, with brittle peelings of the indenter sliding surface and debris accumulating on both sides of the scratch, which is considered substantial damage. The indentation crack critical load of the giant panda sample was between those of the bovid and the dog, and the debris occurred last. This indicates that the substantial damage critical load on giant panda enamel from contact with the diamond head is greater than that of cattle and dogs.

### Friction-wear test

**Figure 12:**
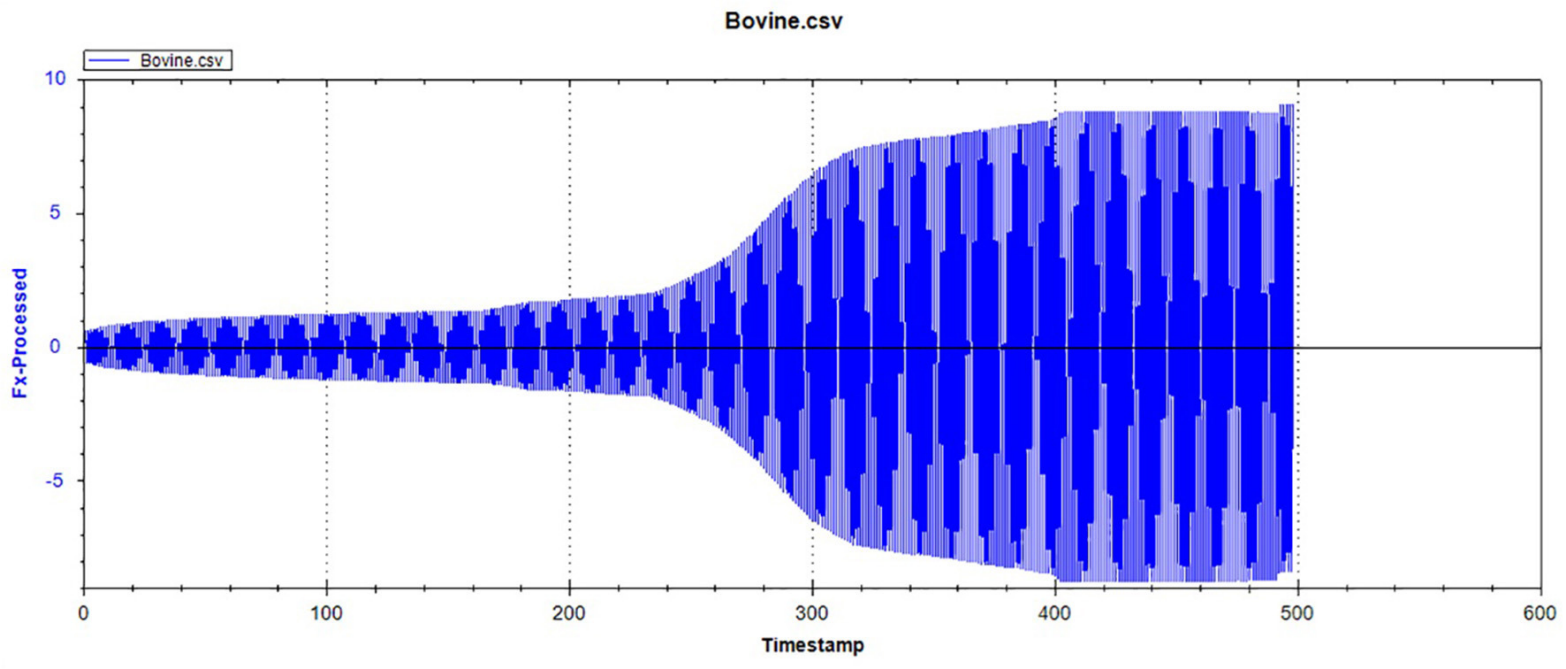
The friction curve of bovine tooth enamel.

**Figure 13:**
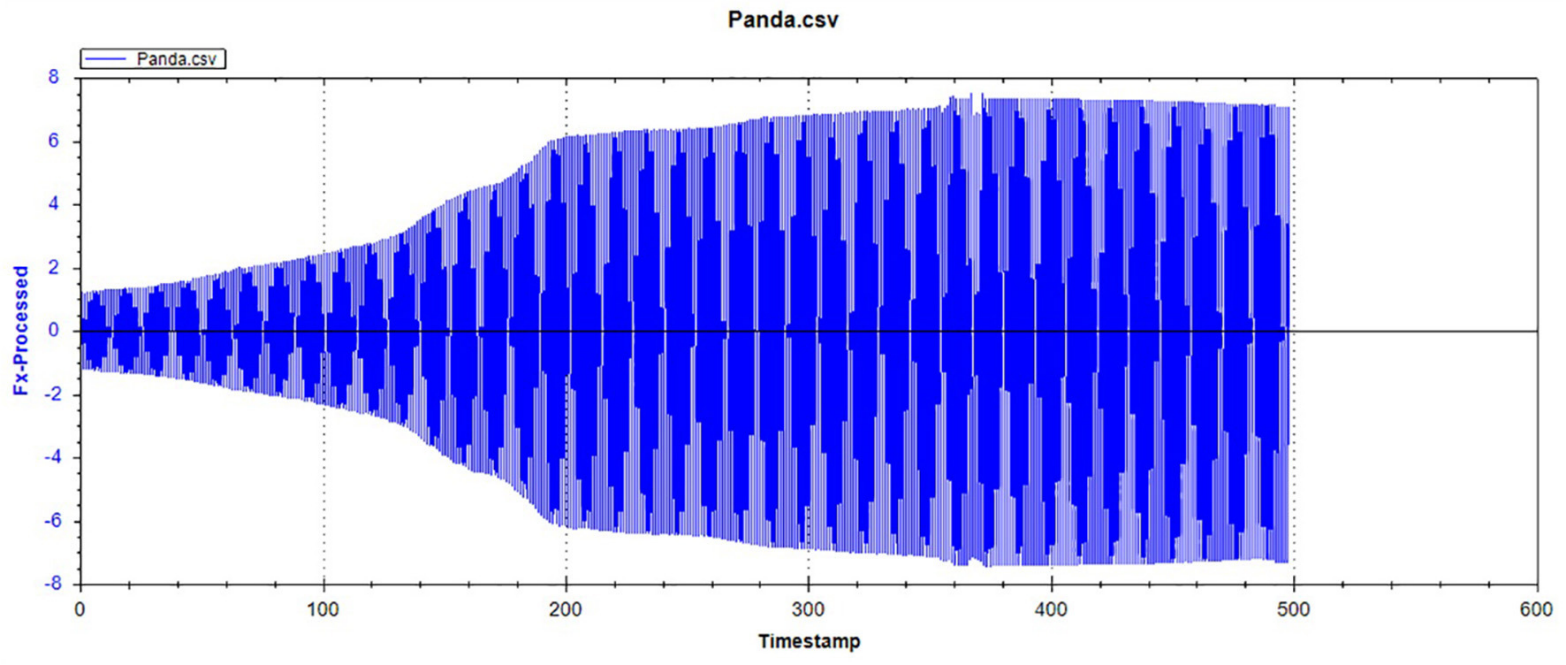
The friction curve of giant panda enamel.

**Figure 14:**
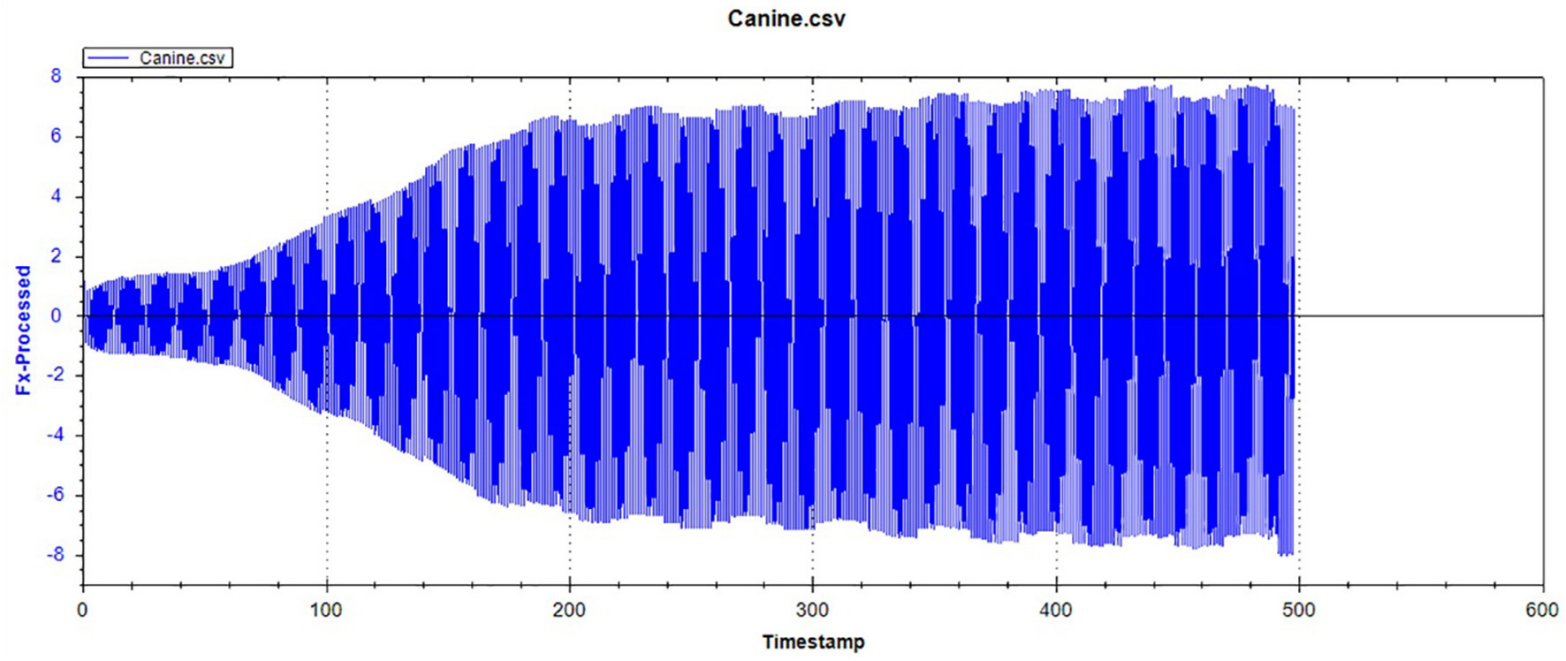
The friction curve of canine enamel.

Figures 12–14 show the tooth enamel friction curves of the bovid, giant panda, and dog under an increasing number of reciprocating cycles. The enamel samples were ground with a silicon nitride ball under a fixed load of 20 mN and lubricated with artificial saliva. It can be seen from Figure 12 that the friction in the bovine enamel remained within a narrow range for 230 s. The friction force changed significantly during the period from 230 to 320 s. The increase rate slowed from 320 to 400 s, and a stable friction force was maintained between 400 and 500 s. According to the giant panda tooth enamel friction curve in Figure 13, it can be seen that the friction changed greatly within the 0–200 s range, which was the period with the most rapid increase. The frictional force slowly increased between 200 and 360s and stabilized between 360 and 500 s. As can be seen from the canine enamel friction curve in Figure 14, the friction increased rapidly from 0 to 190 s before slowing and eventually stabilizing between 19 and 500 s.

**Figure 15:**
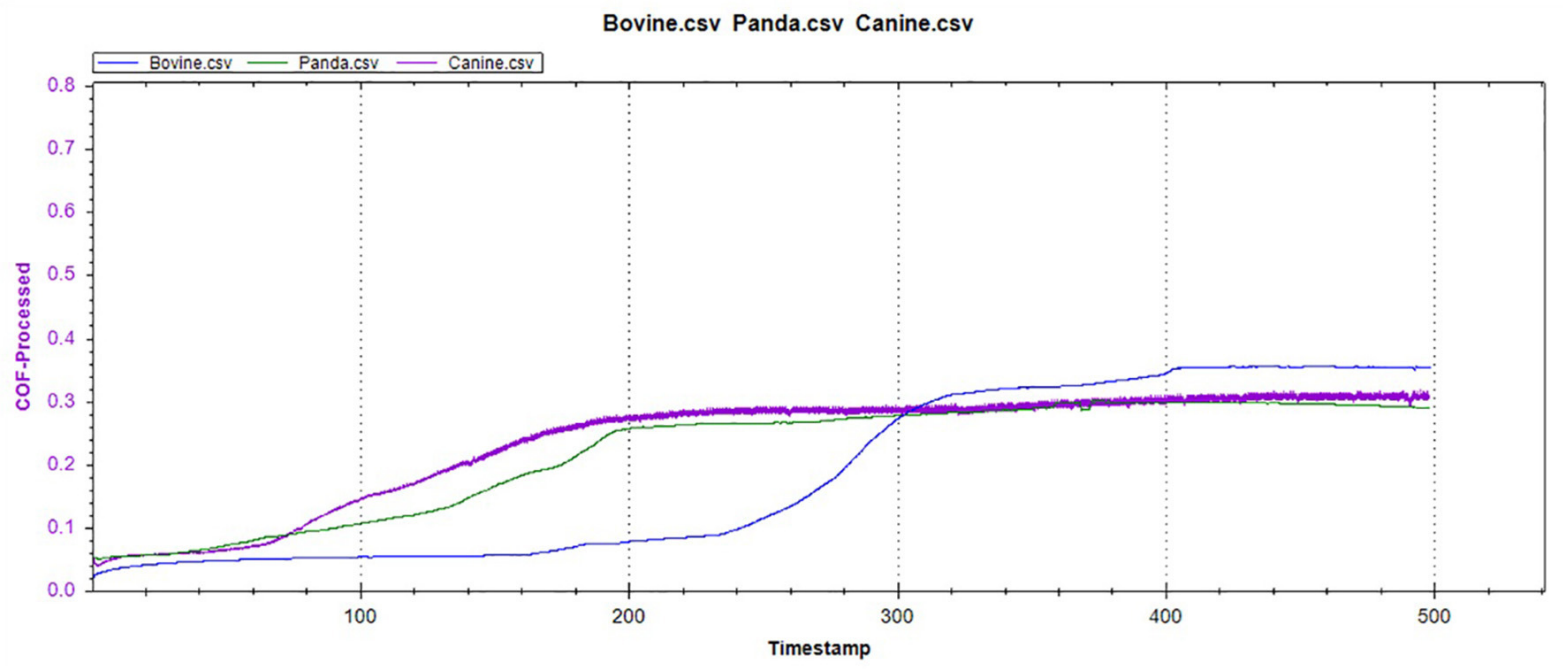
The friction coefficient curve of the canine, giant panda, and bovine teeth enamel.

As presented in Figure 15, all three animals' enamel friction coefficient curves show that the friction change coefficient and friction remained relatively consistent. The friction coefficient change trend between pandas and dogs was similar, and the friction coefficient increased rapidly within the first 200 s. However, the giant panda's enamel friction coefficient was smaller than the dog's and increased slowly to reach the same level as that of the dog—around 200–300 s. The bovid friction coefficient maintained a small increase within their first 230 s before increasing rapidly between 230 and 320 s. After 300 s, the friction coefficient exceeded that of both the dog and panda and was maintained at around 0.36.

## Discussion

The health of a mammal's teeth is critically important to their longevity and quality of life. As permanent teeth last a lifetime, tooth enamel needs to be resistant to wear and tear. Mammalian tooth enamel is mainly composed of hydroxyapatite (HAP), with a mass ratio of approximately 96%, and protein and water, with a mass ratio of 4%. In most mammalian tooth enamel, nano-sized fibrinous HAP crystals are bonded by proteins (mainly hydrophobic amelogenin), forming micron-level basic structural units, which are, in turn, separated and surrounded by a protein-rich sheathing (10). Tooth enamel has unique mechanical properties due to its structural complexity and layering, particularly anti-cracking properties. Compared with its main ingredient, HAP, it satisfies the mechanical requirements of millions of mastications over the organism's lifetime (9, 13–17).

The arrangement of the enamel columns in different thicknesses of giant panda tooth enamel varies; thus, the enamel was divided into outer and inner enamel areas. In the outer enamel region, the enamel columns were arranged neatly and extended to the occlusal surface. The inner enamel zone, however, was composed of alternating cross bands of enamel columns, and the enamel columns were inclined. The two characteristic regions are known as the para-zone and the dia-zone, respectively (18). Where the glaze column was separated both horizontally and vertically, it was distinguished. The Hunter-Schreger Bands (HSBs) were obvious on the side of the tooth, with its width generally consisting of 8–15 enamel columns; the glaze column cross-section was generally hexagonal or quadrangular, and the glaze column diameter increased gradually from the inside outward. The characteristics were as follows: The surface glaze column was 7–9 μm in length and 5–7 μm in diameter; the cross-section shape was hexagonal or irregular; the deep glaze column was 4–5 μm in length and 3–5 μm in diameter, and the cross-section shape was quadrilateral or approximately circular. The number of enamel columns gradually decreased near the enamel dentin boundary or was even completely missing at times, forming enamel without the enamel column structure. At a lower scale, the enamel column displayed continuous changes in the hydroxyapatite microcrystalline nanofiber orientation in the enamel column. The diameter of the crystal fiber constituting the glaze column was about 35 ± 10 nm, with a length greater than 1 μm. These crystalline fibers were arranged neatly in the center of the glaze column and spread to the edge. The enamel sheath thickness was 64 nm, and the boundary was inhomogeneous under TEM. Some crystalline fibers extended from one adjacent glaze pillar to another (9).

Giant panda tooth enamel is very similar to human enamel in terms of mechanical properties, demonstrating superior hardness and Young's modulus (9). Bamboo, however, has higher stiffness, strength, and toughness. Its deformation resistance can reach eight times that of the Chinese Fir, and its tensile strength is six times that of soft steel. Its fracture toughness is also much stronger than ordinary lignocellulose, and it has a higher elastic recovery rate and fracture work in a hydrated state. Compared with bamboo, giant panda tooth enamel toughness is poor, which also emphasizes the key role played by integrating the underlying tissue within the whole tooth and the high hydration of bamboo (19, 20).

During mastication, the enamel is subjected to a mixed-mode stress state. Unlike brittle materials that produce straight or symmetrical cracks when stressed, the cracks in biomaterials tend to be related to their microstructure. When enamel is subjected to stress, the forces are preferentially transmitted and decay along the enamel boundary. The protein components between the enamels break, resulting in longitudinal or transverse cracks or edge peeling with a sharp contact (15, 17, 21, 22).

In the micro-scratch test, accumulated grinding debris appeared first on both sides of bovine enamel. With a higher load, an obvious scratch crack and adjacent cracks on both sides were observed at 306.41 mN, with the adjacent cracks showing a linear extension, which was inconsistent with the shape of the enamel column. Bovine enamel was more similar to the brittle material than the panda and dog; that is, it tended to have higher compressive strength but less impact resistance beyond the critical value.

On the other hand, the adjacent cracks in the giant panda and dog enamel were similar, and conduction was observed along the enamel column boundary. However, the canine crack edge was not as regular as the giant panda's, and the adjacent cracks and debris accumulation appeared almost simultaneously when the load was 222.47 mN. The appearance of debris indicates that the adjacent enamel column has broken laterally. Thus, it can be inferred that the enamel column's overall structure is still damaged. However, the canine tooth enamel reduced the enamel column stress by separating proteins between the enamel columns. Although adjacent cracks appeared earlier in the giant panda's enamel, its hexagonal shape remained regular until the load increased to 334.72 mN, as shown in Supplementary Figures 1, 2. This indicates that the integrity of the giant panda enamel column was maintained more readily than in the dog.

The order in which the scratch and adjacent cracks appeared on both sides of the scratch also proves this point. The scratch crack results from the frictional transverse conduction across the enamel cross-section, while the adjacent crack is formed from the transverse force conduction across the longitudinal section (23, 24). These two types of cracks appeared almost simultaneously, indicating that the ability of bovine enamel to maintain transverse and longitudinal integrity was similar. Scratch cracks appeared earlier in the canine samples, indicating that a transverse fracture of the enamel column was more likely to occur in canine enamel under stress. The giant panda's tooth enamel not only benefits from the fracture of proteins between the enamel columns to ensure the longitudinal integrity of the glaze column, but it also has good transverse fracture resistance. Comparing the giant panda and canine enamel micro-scratch results, it can be seen that the first scratch crack appeared in the canine enamel, while the earliest adjacent crack appeared in the giant panda enamel. Both had a large plastic flow; however, there were differences in the scratch crack location. By comparison, it was found that the dog and giant panda's adjacent cracks were similar to the glaze column shape. Therefore, when plastic flow occurred, a gap appeared between the glaze column and the glaze column. The distance between cracks was less than the glaze column diameter with scratch cracks, likely due to the glaze column cracking. Cracks in the giant panda enamel indentation minimize the damage through crack deflection, distortion, and bridging (9). As the load increases, cracks begin to appear under the indentation. A study by Arsecularatne found that cracks in the lower worn layer of human enamel occurred during the separation of hydroxyapatite fiber and organic phase, followed by the fracture of hydroxyapatite fiber in the surface layer, causing indentation cracks as the load continues to increase under the action of the furrow, both sides of the uplift and debris accumulation (25).

In terms of elastic recovery level, the absolute amount of elastic recovery (i.e., PD-RD value) of the canine sample was significantly greater than that of both the giant panda and bovid, indicating that canine enamel had a greater elastic recovery in the longitudinal direction, which may be a mechanism for compensating for poor longitudinal integrity. By comparing the three Rr curves, we can see that the canine enamel had the highest elastic recovery ability, with the bovine enamel second and panda last. This order is consistent with the food fragility of the three species. Bamboo has higher stiffness, strength, and toughness than the food that dogs eat and most of the plants that cattle eat. The low elastic resilience of giant panda enamel may be related to its adaptive evolutionary strategy for eating bamboo. As its strength is not as good as herbivores, inelastic deformation mechanisms are used more, such as the separation between the enamel columns and sliding along the interface between the enamel columns, the rearrangement and redirection of HAP fragment mechanisms to disperse extrusion stress, and absorption of the impact energy to minimize irreversible damage to the enamel (26–30). The inelastic deformation mechanism is a kind of “conquering the unyielding with the yielding” mechanism.

The giant panda sample had the highest Rd value, suggesting that under the same stress, its tooth enamel was likely to incur deeper scratches. However, a related study has proved that the HAP crystals in giant panda tooth enamel can gradually repair damage under hydration conditions. The discovery of this mechanism suggests that, in the natural state, scratches in giant panda tooth enamel can self-repair to compensate for the shortage of easy-to-leave deep scratches (31).

In the reciprocating friction and wear tests, the three friction coefficient curves of bovid, giant panda, and dog were divided into three stages: low friction coefficient, rapid increase, and maintaining stability. As the tooth surface was polished, the initial plane was relatively smooth, with the friction coefficient being relatively low during the initial stage. As the amount of surface material peeling increased, the roughness constantly increased, from two-body to three-body abrasive wear.

Under the same pressure, the higher the friction coefficient, the more serious the enamel wear. The bovine enamel retained a lower friction coefficient for longer, but once the surface material broke, the friction coefficient increased rapidly, surpassing that of the canine and panda samples. Consequently, even though bovine enamel can withstand friction for more extended periods, once the critical value was breached, the tooth enamel wore away drastically. Therefore, “hard encounter” can be considered the mechanism of bovine enamel against friction and wear during mastication. By enhancing the tooth enamel strength, the normal usage time was prolonged; however, the damage was more serious once the tooth enamel was damaged. The giant panda friction curve was similar to that of the dog, but during the rapidly increasing stage, the friction coefficient grew slower than the dogs and stabilized later. This means that the giant panda's tooth enamel was slightly more resistant to friction and wear than the dog's, which may be related to the structural characteristics mentioned above.

Although giant panda tooth enamel does not have the structural strength of herbivore tooth enamel, it has evolved a series of anti-wear strategies to cope with the heavy burden of bamboo consumption (9, 23, 32). These strategies include prioritizing the integrity of the enamel columns, avoiding direct stress when the enamel is subjected to longitudinal forces, strengthening fracture toughness when the enamel is subjected to transverse forces, and replacing elastic deformation with inelastic deformation to minimize severe friction and wear.

In short, the giant panda's tooth enamel still retains the basic structure of that of carnivores. This comparative study with dogs and cattle confirms that the giant panda's tooth enamel is more similar to that of dogs in terms of cross-sectional shape, friction coefficient, and other physical enamel column properties. Additionally, its wear resistance is inferior to that of cattle. This provides a theoretical explanation for the special giant panda's enamel structure and how its wear resistance could be improved. It provides a direction for follow-up theoretical and experimental research on the giant panda's tooth enamel and its biomaterials.

## Data availability statement

The datasets presented in this study can be found in online repositories. The names of the repository/repositories and accession number(s) can be found in the article/Supplementary material.

## Author contributions

YJ and GL contributed to the conception of the study. YY and YWa performed the experiment. YWu, JL, and ZL contributed significantly to analysis and manuscript preparation. YWu and ST performed the data analyses and wrote the manuscript. CP helped perform the analysis with constructive discussions. All authors contributed to the article and approved the submitted version.

## Funding

This study was supported by the College of Veterinary Medicine at China Agricultural University.

## Conflict of interest

The authors declare that the research was conducted in the absence of any commercial or financial relationships that could be construed as a potential conflict of interest.

## Publisher's note

All claims expressed in this article are solely those of the authors and do not necessarily represent those of their affiliated organizations, or those of the publisher, the editors and the reviewers. Any product that may be evaluated in this article, or claim that may be made by its manufacturer, is not guaranteed or endorsed by the publisher.
