# Supplementary material for: Special architecture and anti-wear strategies for giant panda tooth enamel: Based on wear simulation findings
Source: Front Vet Sci. 2022 Sep 14;9:985733. doi: 10.3389/fvets.2022.985733 (PMC9516319; doi:10.3389/fvets.2022.985733)
Supplement: Supplementary file 1 [file Data_Sheet_1.docx]

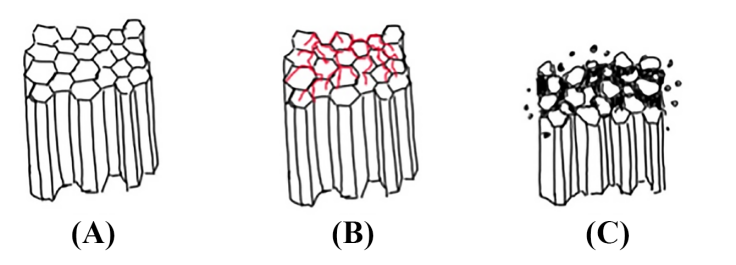


Supplementary Figure 1. Formation of adjacent cracks in canine enamel:(A) Original appearance of enamel column (B) Transmission of force between enamel column（C）The enamel column separates under force, produces fragmentation and forms debris


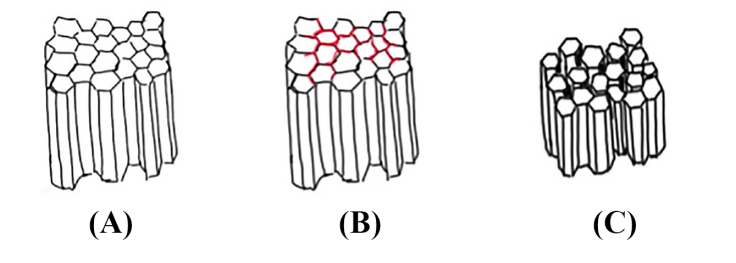


Supplementary Figure 2. Formation of adjacent cracks in giant pandas enamel:(A) Original appearance of enamel column (B) Transmission of force between enamel column（C）The enamel column separates under force, retain integrity and produce no obvious debris
